# An Unusual Case of Elevated Testosterone: When Testosterone by Dialysis Saves the Day

**DOI:** 10.7759/cureus.93597

**Published:** 2025-09-30

**Authors:** Dave K Garg, Run Yu

**Affiliations:** 1 Internal Medicine, University of California Los Angeles David Geffen School of Medicine, Los Angeles, USA; 2 Medicine/Endocrinology, University of California Los Angeles David Geffen School of Medicine, Los Angeles, USA

**Keywords:** assay interference, binding, bioavailable testosterone, lab interpretation, serum total testosterone, sex hormone binding globulin

## Abstract

This case reviews an uncommon clinical scenario in which a markedly elevated total testosterone was detected in the setting of a complaint of hair loss in a male. Clinically, the patient had no other manifestations of elevated testosterone and did not have any notable supplement use that would explain such aberrant values. Due to concerns for potential malignancy, imaging was pursued that failed to show a source. Given the discordance in his lab values and his workup, a testosterone by dialysis was pursued that returned with reassuringly normal range testosterone levels, highlighting the impact of varied testosterone-protein binding affinity among humans and the ongoing value of equilibrium dialysis as a laboratory technique.

## Introduction

Testosterone levels are frequently checked by primary care doctors for common complaints such as low energy, difficulty with weight loss, and hair loss. Medications or substances can interfere with the body’s gonadotropic axis, resulting in lower testosterone levels. For example, commonly used medications such as opioids can suppress testosterone [1]. There has been somewhat controversial data regarding the impact of delta-9 tetrahydrocannabinol (THC), the main psychoactive component of cannabis, on testosterone levels, although the most recent literature seems to favor that marijuana use results in testicular atrophy, altered reproductive hormones, and worsening of semen quality [2]. And while misuse of exogenous bodybuilding or testosterone supplements may unsurprisingly lead to inappropriately high levels of testosterone that can be harmful to a patient, it is uncommon to see apparently naturally high total testosterone levels. Slightly elevated levels may be seen in women with polycystic ovary syndrome or those currently taking contraception due to a corresponding increase in sex hormone binding globulin (SHBG), but it’s rare in men. Normally, when testosterone levels rise in the blood, negative feedback to the hypothalamus and pituitary gland would suppress release of gonadotropin-releasing hormone (GnRH) and luteinizing hormone (LH), thereby reducing testosterone production and keeping levels within physiologic range for their age. When levels beyond this threshold are seen in men, it is particularly concerning as this suggests a possibility of an endogenous overproduction from a new cancerous growth stemming most commonly from the testicles, adrenal glands, or pituitary gland that would require expedited evaluation and treatment.

## Case presentation

A 58-year-old male presented to the endocrinology clinic regarding significantly elevated total testosterone levels. His primary doctor had originally checked testosterone given complaints of hair loss and a receding hair line over the past few years. To the primary doctor’s surprise, his elevated total testosterone was reported >1400 ng/dL (ref 300 - 890 ng/dL).

His medical history was notable for atrial fibrillation and osteoarthritis, but no notable endocrinopathies. On review of symptoms, he denied any obvious asymmetry or change in testicular size. He denied gynecomastia, easy bruising, presence of striae, headaches, and peripheral vision changes. He endorsed lower libido for the past few months but had attributed this to an increase in familial stressors. He followed a healthy diet and stayed active playing soccer; his weight had increased by ~10 lbs in the past few years with his BMI increasing to 28.43 kg/m2. He did report a family history of male pattern baldness in his maternal uncles. His father passed away from colon cancer; to his knowledge, there was no history of testicular cancer. He was advised previously by his primary doctor to start finasteride, but the patient had not yet started the medication given concerns regarding potential side effects. He was taking various supplements including potassium, magnesium, zinc, a general multivitamin, and collagen powder. He also had been taking an unknown liver supplement daily, but this was initiated after the testosterone level was checked. He denied use of any testosterone supplements or other supplements like biotin that may affect the accuracy of testosterone measurements.

**Figure 1:**
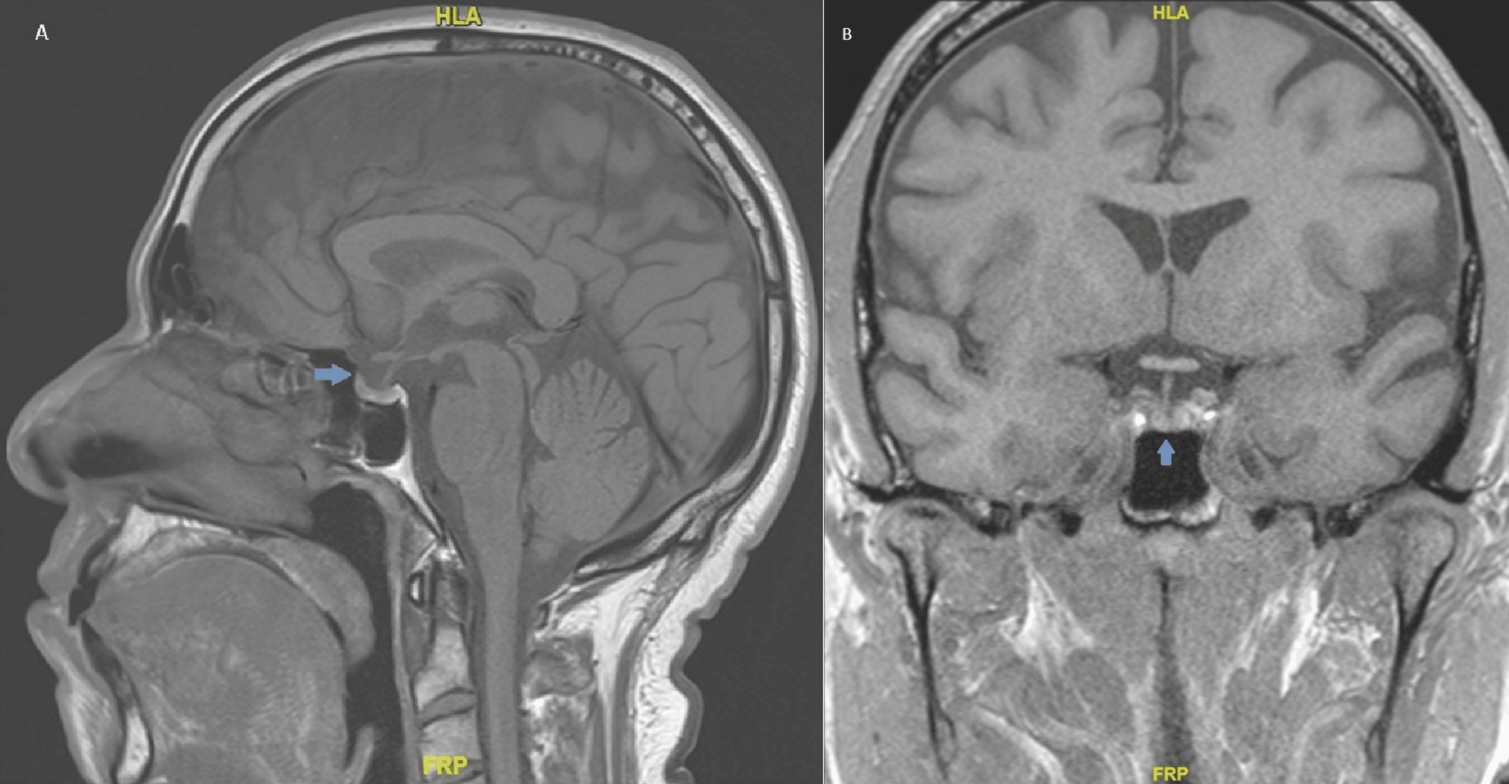
The arrow indicates a normal pituitary MRI in sagittal (A) and coronal (B) sections; no obvious pituitary gland mass.

**Figure 2:**
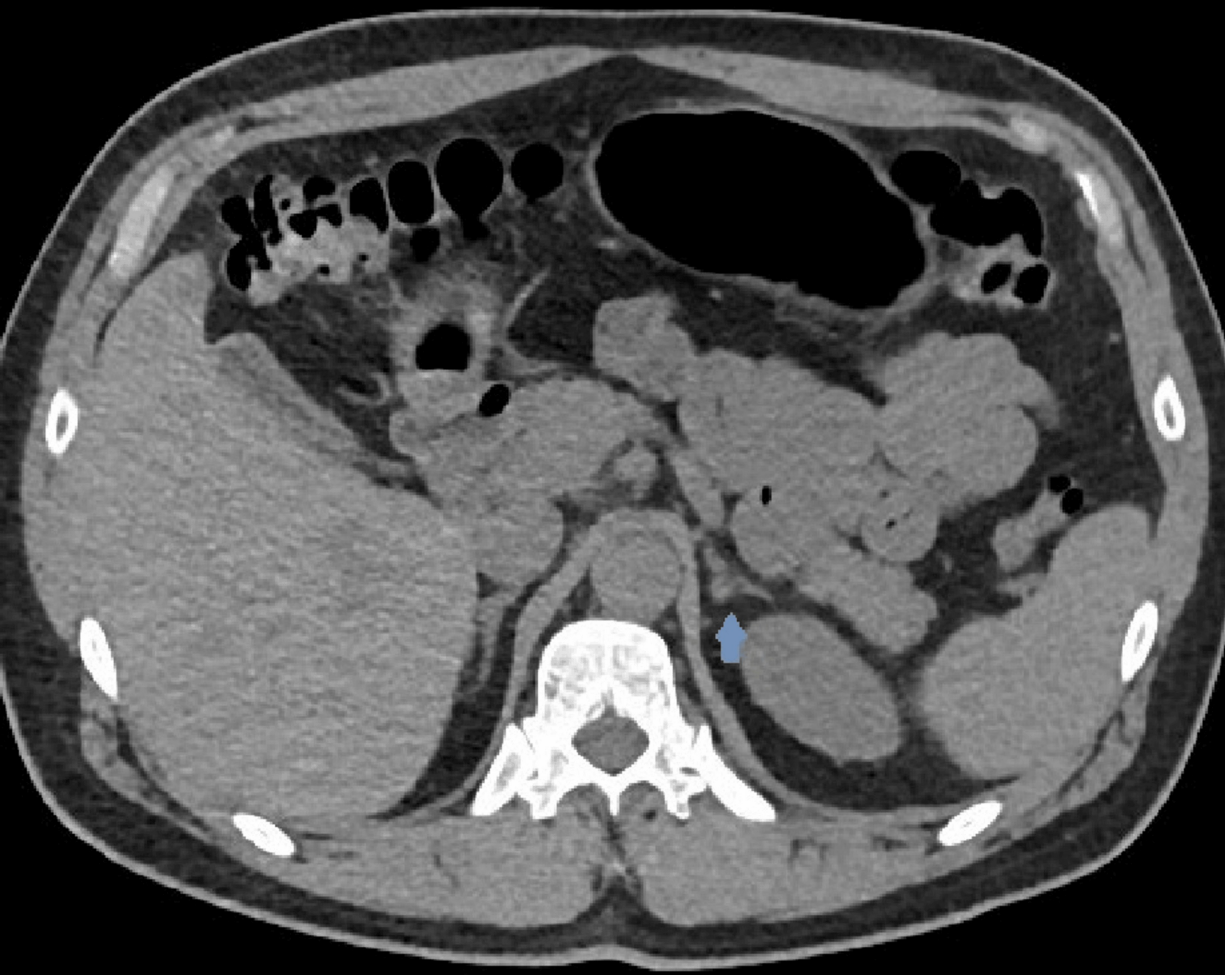
The arrow shows a mildly nodular left adrenal gland on an adrenal CT scan (axial) but without any discrete nodule.

**Figure 3:**
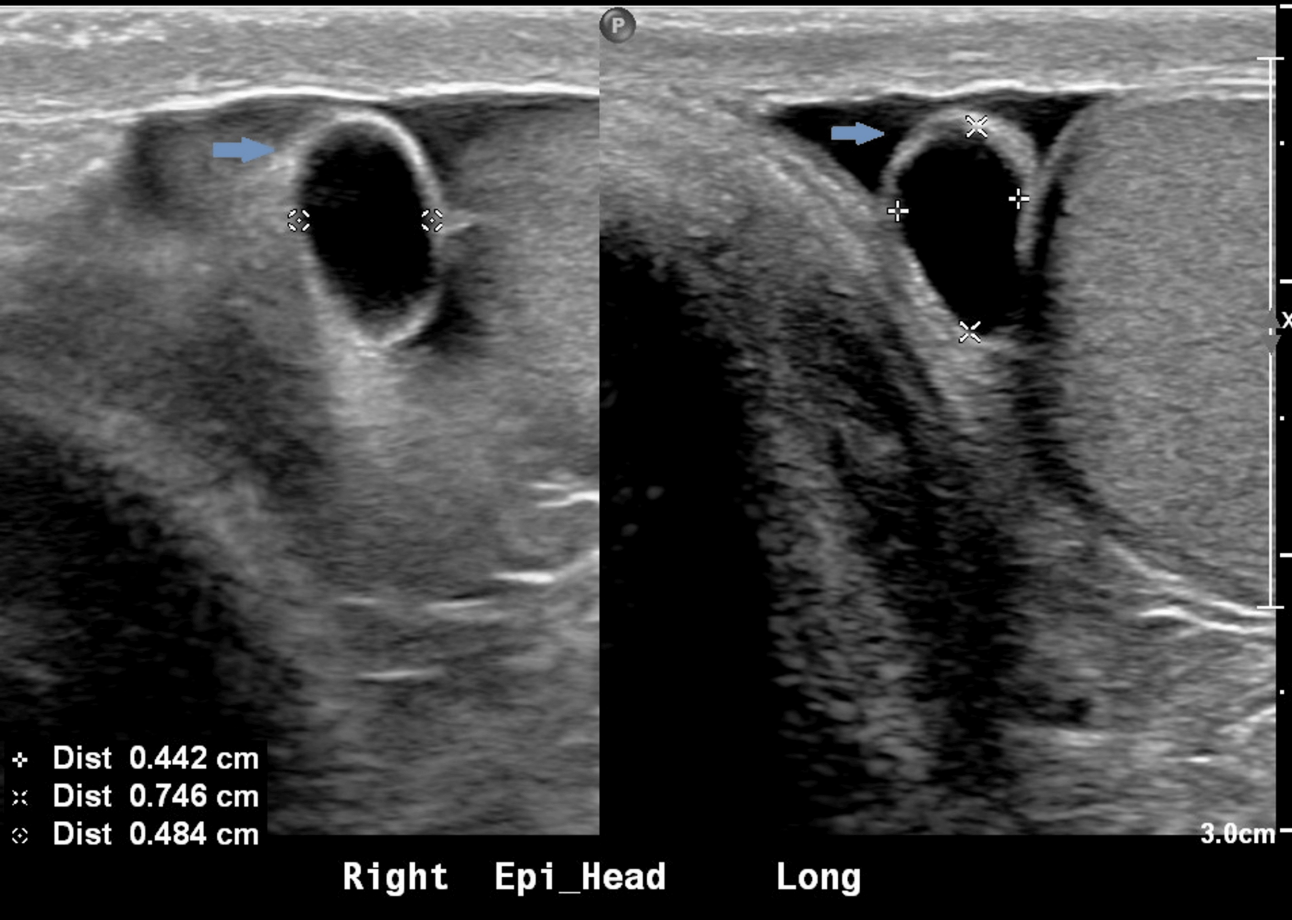
The arrow shows an 8mm right epididymal head cyst but otherwise unremarkable testicular ultrasound.

The patient was advised to hold the unknown liver supplement and levels were rechecked, fasting at 8 am. Repeat testosterone again demonstrated abnormally elevated testosterone >1500 ng/dL. SHBG was measured at 38 nmol/L (ref 19 - 76 nmol/L), in normal range, and bioavailable and free testosterone were reported as elevated at greater than 916 ng/dL (ref 131 - 682 ng/dL) and 329 pg/mL (ref 47 - 244 pg/mL) respectively. Given repeat elevations in testosterone, both total and free, secondary causes were pursued given potentially inappropriately “normal” follicle-stimulating hormone (FSH) 7.8 mIU/mL and LH 3.3 mIU/mL. Pertinent labs are included below (Table 1). A pituitary MRI demonstrated no obvious pituitary gland mass, which was confirmed after discussion with neuroradiology (Figure 1). In the rare case there may be ectopic production, a CT adrenal was done showing a mildly nodular left adrenal gland but without any discrete nodule (Figure 2). A testicular ultrasound showed an 8mm right epididymal head cyst but was otherwise unremarkable (Figure 3). 

**Table 1:** Laboratory values at presentation, repeat assessments, then testosterone by equilibrium dialysis. ED: equilibrium dialysis, LC-MS/MS: liquid chromatography-tandem mass spectrometry

Laboratory Test	Reference	Initial	Repeat #1	Repeat #2	Follow-up
Testosterone, Adult Male	300 - 890 ng/dL	-	>1500	-	-
Sex Hormone Binding Globulin	19 - 76 nmol/L	-	38	-	-
Testosterone, Bioavailable	131 - 682 ng/dL	-	> 916	-	-
Testosterone, Free Calculation	47 - 244 pg/mL	-	> 329	-	-
Testosterone, Percentage Free	1.6 - 2.9 %	-	> 2.2	-	-
Testosterone Total, LC-MS/MS, Males	300.0 - 890.0 ng/dL	-	-	-	342.0
Testosterone Free Adult Male ED/LC-MS/MS	47.0 - 244.0 pg/mL	-	-	-	37.5
Testosterone	200 - 1,000 ng/dL	>1,400	-	>1,400	-
Dehydroepiandrosterone sulfate (DHEA-S)	500 - 4,000 ng/mL	-	757	-	-
Follicle-Stimulating Hormone	1.6-9 mIU/mL	-	-	7.8	-
Luteinizing hormone	2-12 mIU/mL	-	-	3.3	-
Thyroid-Stimulating Hormone	0.3 - 4.7 mcIU/mL	-	2.1	-	-
Hemoglobin A1C	<5.7 %	-	5.7%	-	-

Given the otherwise minimal symptoms endorsed by the patient and negative imaging, a testosterone by dialysis was performed. Testing was collected around 11:30 AM and revealed a total testosterone 342 ng/dL (ref 300-890 ng/dL) and a free adult testosterone 37.5 pg/mL (ref 47-244 pg/mL). This was reassuring and corroborated his clinical story, reinforced by the fact that no major abnormalities were visualized in his pituitary gland, adrenals, or testicles. As the testosterone by dialysis actually showed a lower testosterone level, albeit it was not checked at the optimal time, it was recommended that the patient repeat testing but this was not done.

## Discussion

Testosterone levels were appropriately rechecked in our patient, showing repeat elevation in total testosterone despite the patient being relatively asymptomatic. It is important to remember the significant role binding proteins such as SHBG have in terms of the variation one will see when checking a total testosterone. Common factors such as abnormal thyroid function, insulin resistance, body weight, age, and concurrent liver disease can all impact the production of SHBG, meaning a total testosterone level may no longer accurately reflect a patient’s bioavailable or physiologic testosterone [3]. Table 2 lists common medications and substances known to impact levels of testosterone. And while the majority of testosterone is bound to SHBG or human serum albumin (HAS), free testosterone accounts for only 1-4% of the total testosterone levels.

**Table 2:** Common medications that will affect testosterone levels. SHBG: sex hormone-binding globulin

Increased SHBG concentrations	Decreased SHBG concentrations	Testosterone synthesis inhibition	Increased testosterone
Estrogens	Glucocorticoids	Fluoxetine	Clomiphene
Spironolactone	Progestins	Citalopram	Finasteride
Carbamazepine	Androgenic steroids	Cimetidine	Leuprolide
Phenytoin		Ketoconazole	Tamoxifen
Rifampin		Digoxin	Valproic Acid
		Verapamil	
		Opioids	
		Alcohol	
		Marijuana	
		Anabolic steroids	

To account for possible inaccuracy from various binding proteins and other unlikely but potential interfering substances listed in Table 3 such as biotin [4,5], a free calculated testosterone was checked, but this too was reported as elevated. Given these concerning findings, an etiology for the high testosterone was pursued resulting in additional testing including an MRI pituitary and an adrenal CT scan, both of which returned normal. In the off chance there was a primary source, a testicular ultrasound was also done that was unremarkable. The free testosterone is helpful in getting a sense of a patient’s physiologic testosterone levels in most cases, but it’s notable that free testosterone is often a calculated value based on various equations that depend on correct estimates of the association constants and stoichiometry for binding of testosterone to SHBG and albumin [6]. There is literature that describes a variety of SHBG variants, for example variants with lower affinity for testosterone with a higher equilibrium dissociation constant than the wild-type SHBG [7,8].

**Table 3:** Medications and substances that may interfere with testosterone assays. Table was created independently by the authors using information from the cited sources [4,5].

Endogenous compounds	Medications	Supplements
Heterophile antibodies	Anabolic steroids	Biotin
Sex Hormone-Binding Globulin (SHBG)	Norethisterone (synthetic progestin)	St. John’s Wort
Dehydroepiandrosterone (DHEA)	Danazol	N-acetyl cysteine (NAC)
Dehydroepiandrosterone Sulfate (DHEA-S)	Mifepristone	Maca
		Fluorescein

**Figure 4:**
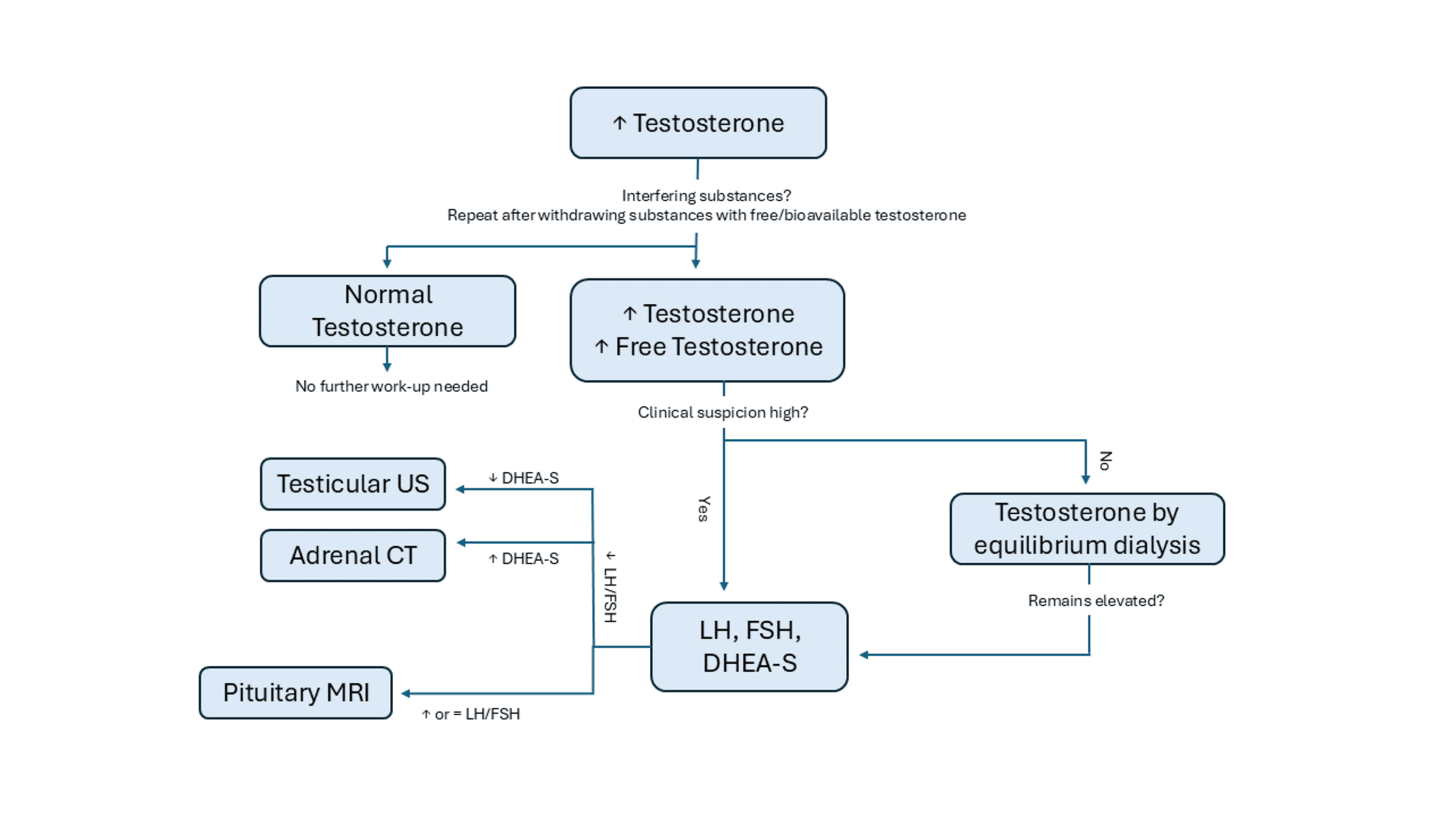
Diagnostic algorithm for elevated testosterone. LH: luteinizing hormone, FSH: follicle-stimulating hormone, DHEA-S: dehydroepiandrosterone sulfate, US: ultrasound Figure was created independently by the authors.

While it is more expensive and labor-intensive, one of the advantages of testosterone by dialysis is that it is not subject to the same reliance on these estimated binding affinities of testosterone to SHBG and HSA. This is because the methodology involves the dialysis of a serum or plasma sample whereby the protein-bound testosterone is retained while the free testosterone permeates across a cellulose membrane and values can then be directly measured using a liquid chromatography-tandem mass spectrometer (LC-MS/MS), as was the case for our patient, or indirectly using a tracer [4]. Given the extremely high total and calculated free testosterone, compared to the very normal and arguably lower free/total testosterone by dialysis, it would suggest that our patient most likely had a SHBG or albumin variant with altered binding affinity, resulting in inaccurate readings when using calculated values based on assumed binding constants and measurement of the total testosterone via immunoassay. See Figure 4 for a proposed algorithm for the evaluation of elevated testosterone.

## Conclusions

In summary, we present a case of a markedly elevated testosterone level detected via immunoassay in a largely asymptomatic male patient without any radiologic evidence to suggest a source. Considering the potential for less accurate measurement of testosterone with the immunoassay method due to altered affinity of testosterone to binding proteins like SHBG or albumin, a testosterone by equilibrium dialysis was performed which resulted in a near normal value. This case report highlights a couple key factors. There are SHBG and albumin variants that can result in different binding affinities and therefore potential false positive and negative values of testosterone, which a provider should question if the clinical context seems to suggest otherwise. While more costly and time-intensive, the testosterone by equilibrium dialysis remains the gold standard method for measuring testosterone and should be utilized earlier in these unusual scenarios of unexplained, or rather peculiar, elevations in testosterone.
